# Supplementary material for: Shared Treg dysregulation underlies psoriasis vulgaris and atopic dermatitis through the PHF19-PRC2 epigenetic axis
Source: Front Immunol. 2026 Aug 20;17:1932788. doi: 10.3389/fimmu.2026.1932788 (PMC13538097; doi:10.3389/fimmu.2026.1932788)
Supplement: Supplementary file 3 [file DataSheet3.zip › Additional files/Additional file 6/25.PHF19.forest.pdf]

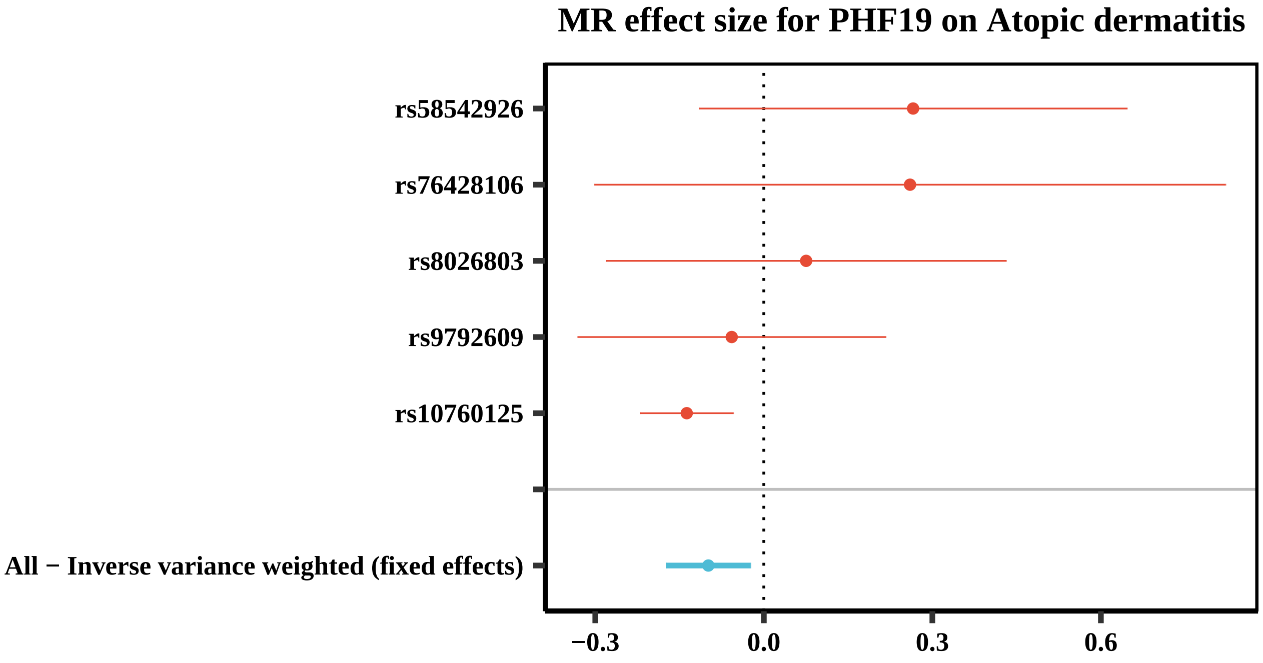
MR effect size for PHF19 on Atopic dermatitis
rs58542926
rs76428106
rs8026803
rs9792609
rs10760125
All − Inverse variance weighted (fixed effects)
−0.3
0.0
0.3
0.6
